# Quaternary climatic oscillations shaped the demographic history and triggered intraspecific divergence of *Rhododendron shanii*, a mid-montane endemic in eastern Asia

**DOI:** 10.3389/fpls.2025.1740252

**Published:** 2026-01-12

**Authors:** Yong Deng, Zhen Li, Yingfeng Hu, Zhizhong Li, Siyu Zhang, Kun Liu, Jianwen Shao

**Affiliations:** 1 College of Life Sciences, Anhui Normal University, Wuhu, Anhui, China; 2 Anhui Forest Survey and Planning Institute, Hefei, Anhui, China; 3 The Anhui Provincial Key Laboratory of Biodiversity Conservation and Ecological Security in the Yangtze River Basin, Anhui Normal University, Wuhu, Anhui, China; 4 Collaborative Innovation Center of Recovery and Reconstruction of Degraded Ecosystem in Wanjiang Basin, Anhui Normal University, Wuhu, Anhui, China; 5 College of Civil and Architecture Engineering, Chuzhou University, Chuzhou, Anhui, China

**Keywords:** demographic history, ecological niche modeling, gene load, genomeresequencing, Sky islands

## Abstract

Mountainous regions often serve as critical biodiversity hotspots. In mid-altitude mountains, populations may be more vulnerable to climate-driven fluctuations than those in alpine regions due to limited capacity for elevational range shifts. However, empirical studies on how past climatic changes shaped the demographic history of organisms in the mid-mountains remain scarce, particularly those utilizing genomic data. Here, we conducted population genomic analyses of *Rhododendron shanii*, an endemic species in the Dabie Mountains of eastern Asia. Combined with species distribution modeling, our demographic analyses indicate that this species underwent glacial expansion during Quaternary cooling periods but experienced three distinct population bottlenecks over the past 0.4 million years, all coinciding with interglacial warm periods. Its population size has continuously declined throughout the Holocene as temperatures rose. Significant genetic differentiation has occurred among populations inhabiting different mountaintops despite their highly restricted distribution. Notably, warm conditions during the last interglacial period (0.12–0.13 Mya) triggered the divergence between the southern lineage (S: TJ, SBG, DZJ) and the northern lineage (N: THJ, BMJ, DYJ). Compared to closely related species, *R. shanii* currently exhibits a high inbreeding rate yet maintains relatively high genetic diversity and low genetic load. This unique genetic signature is likely linked to its recent rapid population contraction. Collectively, our findings demonstrate how Quaternary climatic oscillations and mid-mountain topography shaped the demographic trajectories and genomic landscape of *R. shanii*, providing new insights into the formation and vulnerability factors of biodiversity within mid-elevation sky island systems under global warming scenarios.

## 1 Introduction

**Figure 1:**
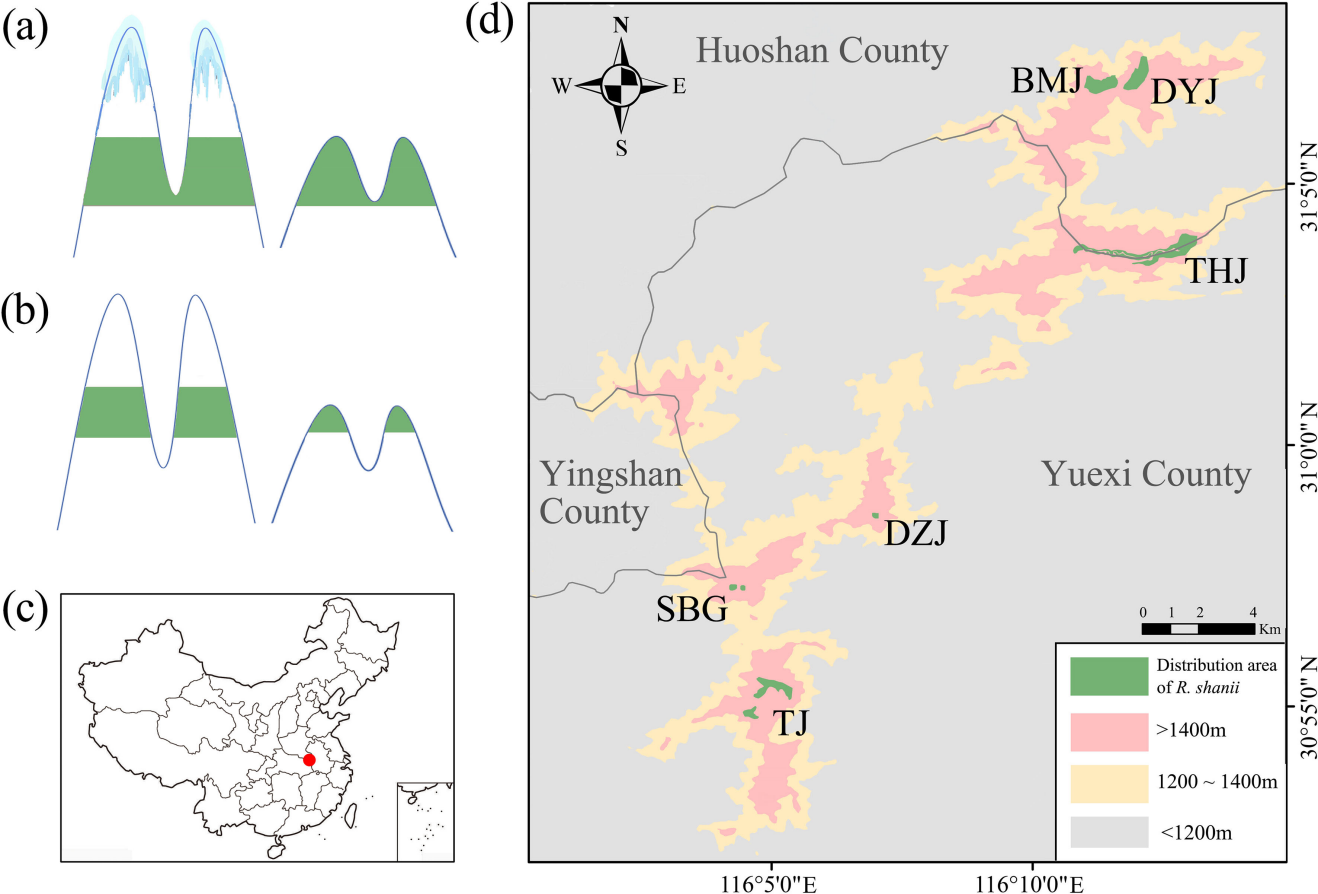
Locations and population distribution diagram of *R. shanii.***(a)** Population distribution during glacial periods. **(b)** Population distribution during inter-glacial periods. Green bands represent species distribution. Diagrams **(a, b)** indicate that limited elevational space in low mountains leads to rapid population decline during climatic fluctuations. **(c)** Location of the Dabie Mountains in China. The red dot marks the Dabie Mountains, the distribution area of *R. shanii.***(d)** Distribution and populations of *R. shanii*.

Mountainous systems are widely recognized as critical biodiversity hotspots that maintain exceptional species richness and harbor disproportionate levels of endemic diversity (Myers et al., 2000). The origin and evolution of biodiversity in mountains are generally dependent on their topographical landscapes and associated historical climatic changes (Liu et al., 2014; Rahbek et al., 2019; Sun et al., 2017). Pronounced elevational gradients and topographic complexity inherent to mountainous terrains generate both habitat heterogeneity and population fragmentation, facilitating evolutionary radiation and *in situ* speciation (Hughes and Atchison, 2015; Muellner-Riehl, 2019; Rahbek et al., 2019). In addition, during Quaternary climate oscillations, mountainous ecosystems provided crucial refugia, enabling populations to track suitable habitats through elevational shifts, thereby enhancing their persistence and survival (Abbott et al., 2000). These cyclical range shifts induced recurrent population fragmentation and secondary contact along topographic gradients, thereby establishing an "evolutionary pump" mechanism that promoted divergence and ultimately speciation (Petit et al., 2003; Sun et al., 2017; Wu et al., 2022). Mountainous regions, with their unique biodiversity aggregations, serve as natural laboratories for studying the mechanisms driving biodiversity initiation and maintenance across temporal and spatial scales (Mao et al., 2021; Zhang et al., 2019b). Although biogeographic studies on this topic are not uncommon, they have primarily focused on typical alpine biodiversity hotspots (Chen et al., 2019; Ren et al., 2017; Zhang et al., 2019b). The effects of Quaternary climatic oscillations on species demography vary substantially across regions and continents (Hewitt, 2004; Ren et al., 2017). For organisms in mid-elevation mountains, particularly cold-adapted plants with narrow elevational ranges, limited opportunities for upward range shifts hinder their ability to track suitable habitats and maintain stable populations. Consequently, their population sizes are more vulnerable to climate-driven fluctuations than those in alpine regions (**Figures 1a, b**). However, how demographic changes during range shifts shape spatial genetic variation within and among these mid-elevation populations remains poorly understood.

The Dabie Mountains, situated at mid-latitudes (30.1°–31.8°N) in eastern China, comprise a chain of ancient, isolated, low- to mid-elevation massifs (**Figure 1c**), with Baima Peak (1,777 m a.s.l.) as the highest point. This region serves as a critical water source conservation area for the middle-lower reaches of the Yangtze and Huai Rivers and constitutes a biodiversity hotspot in eastern Asia (Tang et al., 2006). It harbors rich species diversity, including numerous endemic plants and animals, such as *Pinus dabeshanensis*, *Dendrobium huoshanense*, *Pachyhynobius shangchengensis*, and *Moschus anhuiensis* (Shen, 1995; Zhang et al., 2016b; Pan et al., 2019b). Ecologically, this region lies within the transitional zone between subtropical evergreen broad-leaved forest and warm-temperate deciduous broad-leaved forest. Palynological evidence indicated that temperate deciduous forests, dominant in northern China (30°N–42°N) today, retreated southward to 25°N–30°N during the Last Glacial Maximum (Harrison et al., 2001). These Quaternary climatic oscillations likely triggered profound demographic shifts in the Dabie Mountains flora, significantly reshaping its genetic architecture and contributing to biodiversity generation. For instance, Pan et al. (2019a) used mtDNA data to demonstrate that the regional endemic salamander *Pachyhynobius shangchengensis* has diversified into multiple highly divergent lineages, potentially representing cryptic species. Nevertheless, empirical studies examining the detailed demographic history and genetic consequences of these climatic oscillations on plants across this biodiversity hotspot remain limited.

*Rhododendron shanii* Fang (Ericaceae), an endemic species of the Dabie Mountains in eastern China, exhibits a highly restricted distribution confined to just six discrete mid-elevation localities (>1400 m a.s.l.) in western Yuexi County and along the Yuexi-Huoshan border (**Figure 1d**). As a constructive species typically found in mountaintops or near-summit vegetations, its distribution spans three southern peaks: Tuojian (TJ), Duozhijian (DZJ), and Shibigou (SBG), and three northern peaks: Tianhejian (THJ), Baimajian (BMJ), and Duoyunjian (DYJ) (**Figure 1d**; Zhao et al., 2010, Zhao et al., 2012). This relictual distribution across isolated mountain summits, coupled with its narrow climatically sensitive elevational range, makes *R. shanii* an ideal model for investigating how historical climate fluctuations and regional topography shaped demography and genetic patterns in mid-elevation mountain ecosystems.

In this study, we performed population genomic analyses on 48 individuals sampled from all six extant populations for *R. shanii* (**Table 1**). Utilizing this genomic resource, we assessed genome-wide genetic diversity, population structure, and demographic history. Specifically, we aimed to: (1) reconstruct the phylogeographic structure of *R. shanii* within the Dabie Mountains and identify drivers of intraspecific divergence; (2) retrace its detailed demographic trajectory; and (3) evaluate the impact of Quaternary climate fluctuations on its population dynamics using species distribution modeling (SDM) integrated with approximate Bayesian computation (ABC). To place these findings in a broader evolutionary context, we further compared *R. shanii* with two related species—the critically endangered, narrowly distributed *R. griersonianum* and the non-threatened, widespread *R. delavayi*—to examine how demographic history influences genetic diversity and the accumulation of deleterious mutation. Collectively, our findings provide key insights into the evolutionary consequences of climate change in mid-elevation ecosystems and inform conservation strategies for endemic mountain species.

**Table 1:** The sample location information of R. shanii.

Population	Locations (Longitude, Latitude)	Altitude (m)	Population size	Peak altitude of mountain (m)	Distribution area (hectares)
TJ	Tuojian of Hetu, Yuexi County (116.088°E, 30.922°N)	1,600–1,730	ca. 2,000	1,751	52.4
SBG	Shibigou of Hetu, Yuexi County (116.057°E, 30.954°N)	1,650–1,680	ca. 150	1,690	2.4
DZJ	Duozhijian of Yaoluoping, Yuexi County (116.121°E, 30.975°N)	1,630–1,670	ca. 100	1,721	4.5
THJ	Tianhejian at the border of Yuexi and Huoshan Counties (116.192°E, 31.059°N)	1,500–1,730	ca. 2,100	1,766	85.1
BMJ	Baimajian of Mozitan, Huoshan County (116.181°E, 31.113°N)	1,600–1,760	ca. 500	1,777	31.6
DYJ	Duoyunjian of Mozitan, Huoshan County (116.209°E, 31.116°N)	1,600–1,750	ca. 300	1,763	19.9

## 2 Materials and methods

### 2.1 Sample collection and genome sequencing

*Rhododendron shanii* is a small tree (1.5–6.5m high) with thick, leathery and entire leaves. A terminal racemic umbel inflorescence composed of 10–14 light purple red flowers, about 4 cm in diameter (Wu et al., 2005). The flowering period is from May to June, and the capsules mature from September to October. All six known populations were sampled (**Figure 1d**; **Table 1**). Fresh leaves of eight individuals per population (n=48 total) were collected and stored in dry silica gel for genome resequencing. Genomic DNA was extracted using a modified cetyltrimethylammonium bromide (CTAB) method (Agbagwa et al., 2012). All sequencing and library construction were performed by Novogene Biotechnology Co., Ltd. (Beijing, China) according to standard procedures. Raw data were processed by fastp v.0.23.2 (Chen, 2023) with default parameters.

### 2.2 SNP calling and filtering

All clean data were aligned to the reference genome (*R. shanii*, the accession number of CNGBdb Genome database: GWHGGFK00000000) using BWA-MEM v.0.7.17 (Li, 2009), followed by duplicate marking with Picard MarkDuplicates (Broad Institute) and sorting using samtools v.1.13 (Danecek et al., 2021). Single-sample variant calling was performed via GATK v.4.0.5.1 HaplotypeCaller to generate genomic variant call format (GVCF) files. Joint genotyping was then conducted using GATK CombineGVCFs and GenotypeGVCFs to integrate variants across all samples. SNPs were extracted using SelectVariants under default parameters. Initial SNP filtering was applied with GATK VariantFiltration using the criteria: QD < 2.0 || FS > 60.0 || MQ < 40.0 || QUAL < 30.0 || MQRankSum < -12.5 || ReadPosRankSum < -8.0. Subsequent filtering steps included: (i) Removal of non-biallelic loci; (ii) Exclusion of sites with depth less than one-third of the mean coverage across all samples; (iii) Application of population-level filters via vcftools v.0.1.16 (–maf 0.05 –max-missing 0.8) to retain SNPs with minor allele frequency ≥5% and genotype call rate ≥80%; (iv) Pruning of LD sites using Plink v.1.9 (–indep-pairwise 50 10 0.2) to remove variants with pairwise r² > 0.2 within 50-SNP sliding windows (Danecek et al., 2011; Purcell et al., 2007). Raw sequencing data of 31 *R. griersonianum* and 30 *R. delavayi* from GenBank (**Supplementary Table 1**) were processed identically against their respective reference genomes (*R. griersonianum*, *R. delavayi*, Ma et al., 2021), using the aforementioned alignment and SNP calling pipeline. The resulting high-confidence VCF files were retained for downstream population genetic analyses.

### 2.3 Genome-wide genetic diversity and population structure analysis

Genome-wide genetic diversity indices *θ*_w_ (Watterson’s estimator) and *θ*_π_ (nucleotide diversity) of the populations and lineages of *R. shanii* and the two related species (*R. griersonianum* and *R. delavayi*) were calculated using ANGSD v.0.940 (Korneliussen et al., 2014). Neutrality tests were performed by estimating Tajima’s *D* via ANGSD’s allele frequency spectrum module (Tajima, 1989). Additionally, the unfolded site frequency spectrum (*SFS*) for *R. shanii* and its two lineages was also generated using ANGSD. Pairwise genetic differentiation (*F*_ST_) among populations was quantified using vcftools v.1.16 (Danecek et al., 2011), employing a sliding window approach (window size: 20 kb; step size: 10 kb) and correlate it with geographic distance. The filtered SNP dataset was converted to BED format for downstream analyses. Population genetic structure was inferred through ADMIXTURE v.1.3.0 (Alexander et al., 2009) with *K*-values ranging from 2 to 6. The optimal *K* was determined by minimizing cross-validation (CV) error. Principal component analysis (PCA) was conducted using Plink v.1.9 (Purcell et al., 2007), and a neighbor-joining tree (NJ) was constructed using MEGA v.11 (Tamura et al., 2021) with the p-distance method and the clade supports were calculated using 1000 bootstraps. To investigate potential gene flow events among five redefined groups of *R. shanii* (with populations BMJ and DYJ merged into a single group based on admixture), this study employed the ABBA-BABA statistical framework using Dsuite v0.5 with the default process (Malinsky et al., 2021).

### 2.4 Estimates of genetic loads and deleterious mutations

LD decay analysis was performed using PopLDdecay v.3.41 with pairwise r² statistics calculated in 100-kb windows (Zhang et al., 2019a). The LD decay rates of these three *Rhododendron* species were compared via nonlinear regression of r² against physical distance. The location of equilibrium r² was 5% of the difference between the maximum and minimum r², and the physical distance of this location. The proportion of homozygous genotypes (A1A1/ A2A2) relative to total SNPs was extracted from Plink v.1.9 outputs. Runs of homozygosity segments were identified with Plink under the following parameters (–homozyg-density 10 –homozyg-gap 100 –homozyg-kb 100 –homozyg-snp 10 –homozyg-window-het 1 –homozyg-window-missing 5 –homozyg-window-snp 50), and further compared the whole genome length to obtain *F*_ROH_ values.

To assess genome-wide mutation loads in *R. shanii*, *R. griersonianum*, and *R. delavayi*, we extracted all variable sites separately for each individual. These variants were annotated using SnpEff v.4.3 (Cingolani et al., 2012) and classified as synonymous mutations, nonsynonymous mutations, or putative loss-of-function (LoF) mutations (including stop-gain, frameshift, and splice-site variants). The functional impact of these variants was assessed using SIFT4G (Vaser et al., 2016), employing species-specific annotation databases constructed from annotation information for each species and UniRef90 datasets via the make-SIFT-db-all.pl script. Variants with SIFT score <0.05 were classified as deleterious mutations (DEL, mildly deleterious mutations). The number of synonymous mutation sites was determined based on dual verification by SnpEff and SIFT4G. Individual genetic loads were quantified as LoF load (LoF/Synonymous ratios) and DEL load (DEL/Synonymous ratios). Species-level differences in genetic load, *F*_ROH_, and homozygosity ratios were statistically compared using Kruskal-Wallis tests.

### 2.5 Demographic inference and simulations

Population demographic trajectories were reconstructed using FitCoal v.1.1 (Fast Infinitesimal Time Coalescent process, Hu et al., 2023), a coalescent-based framework optimized for inferring historical effective population size (*Ne*) from the unfolded *SFS*. The analysis was performed with the following parameters (-mutationRate 0.41e-5 -generationTime 10 -genome Length 687,733 -noEG -noED -numOfIntervals 45). Bootstrap resampling with 1,000 independent simulations quantified parameter uncertainty. *Ne* estimates were averaged across replicates at 10,000-year intervals and visualized as a continuous time series spanning 10–1,000 ka before present (BP). We also employed two other approaches, i.e., PSMC v.0.6.5 (Pairwise Sequentially Markovian Coalescent, with parameter “-N25 -t15 -r5 -p ‘4+25*2+4+6’”, Li and Durbin, 2011) and Stairway Plot v.2.0 (with default parameter except for mutation rate, Liu and Fu, 2015), to infer the demographic history of *R. shanii* according to the instructions. The mutation rate μ = 0.41 × 10–^8^ base/generation estimated by Ma et al. (2021) was applied in this study.

Then the divergence time between two *R. shanii* lineages was estimated by MSMC2 (with parameter 10 yr/generation and mutation rate of 0.41 × 10–^8^ base/generation, Schiffels and Wang, 2020). Population size histories and split time of two lineages of *R. shanii* were also inferred by SMC++ v.1.15.4 (with parameter “–spline cubic –knots 100 –timepoints 10000 2000000 –cores 24 0.41e-8”, Terhorst et al., 2017). Finally, Fastsimcoal 2 (Excofffier et al., 2021) was used to simulate and infer the demographic dynamics and the split time of two *R. shanii* lineages. The scenarios and corresponding prior distributions of the Fastsimcoal 2 parameters (e.g., lineage divergence, lineage divergence times, *Ne*) were set according to results from the above demographic analyses (e.g., SMC++ and FitCoal). In total, 15 demographic models were evaluated (see **Supplementary Figure 1** for details). Each model underwent 100 independent likelihood maximizations to mitigate local optima risks. Model selection prioritized the Akaike Information Criterion (AIC) and Akaike weights, with the highest-weight model identified as optimal (Wagenmakers and Farrell, 2004). Parameter confidence intervals for the best-fit model were derived from 100 parametric bootstrap replicates, each iterated 100 times to address model fit uncertainty and coalescent stochasticity (Wang et al., 2016).

### 2.6 Niche reconstruction

Ecological niche modelling (ENM) was carried out using the program MaxEnt v.3.4.4 (Phillips and Dudık, 2008) to simulate the possible suitable habitats of *R. shanii* under different climate backgrounds using climatic data and location information of the known distribution localities. Four periods climate data, i.e., Last Interglacial (LIG, 0.14–0.12 Mya), Last Glacial Maximum (LGM, 0.021–0.018 Mya), current (1970–2000), and future (2080–2100, SSP5-8.5), were sourced from the WorldClim database (http://www.worldclim.org) and PaleoClim database (http://www.paleoclim.org/) (Brown et al., 2018; Poggio et al., 2018). A standard 30-arcsecond resolution was applied to all periods.

To mitigate multicollinearity effects, we first extracted climate variables at all occurrence points using ArcGIS v.10.6. Pairwise Pearson correlation coefficients were calculated, and variables with |r| > 0.8 were excluded, simultaneously based on Jackknife tests of variable contribution rates in MaxEnt v.3.4.4 (Pearson et al., 2013). Model calibration adhered to the following protocol: (i) Data partitioning: 70% training set and 30% testing set; (ii) Validation: 10-fold cross-replication; (iii) Convergence threshold: 10^-5^; (iv) Maximum iterations: 5,000; (v) Other parameters: Default settings. Model performance was evaluated using the Area Under Curve (AUC), with values >0.9 indicating high predictive accuracy. The area of the suitable distribution zone was generated by reclassifying MaxEnt’s continuous output in ArcGIS into four discrete categories: unsuitable (0–0.2), marginally suitable (0.2–0.5), moderately suitable (0.5–0.7), and highly suitable (0.7–1.0). Habitat suitability maps were generated by continuous suitable categories.

## 3 Results

### 3.1 Resequencing and population structure

**Figure 2:**
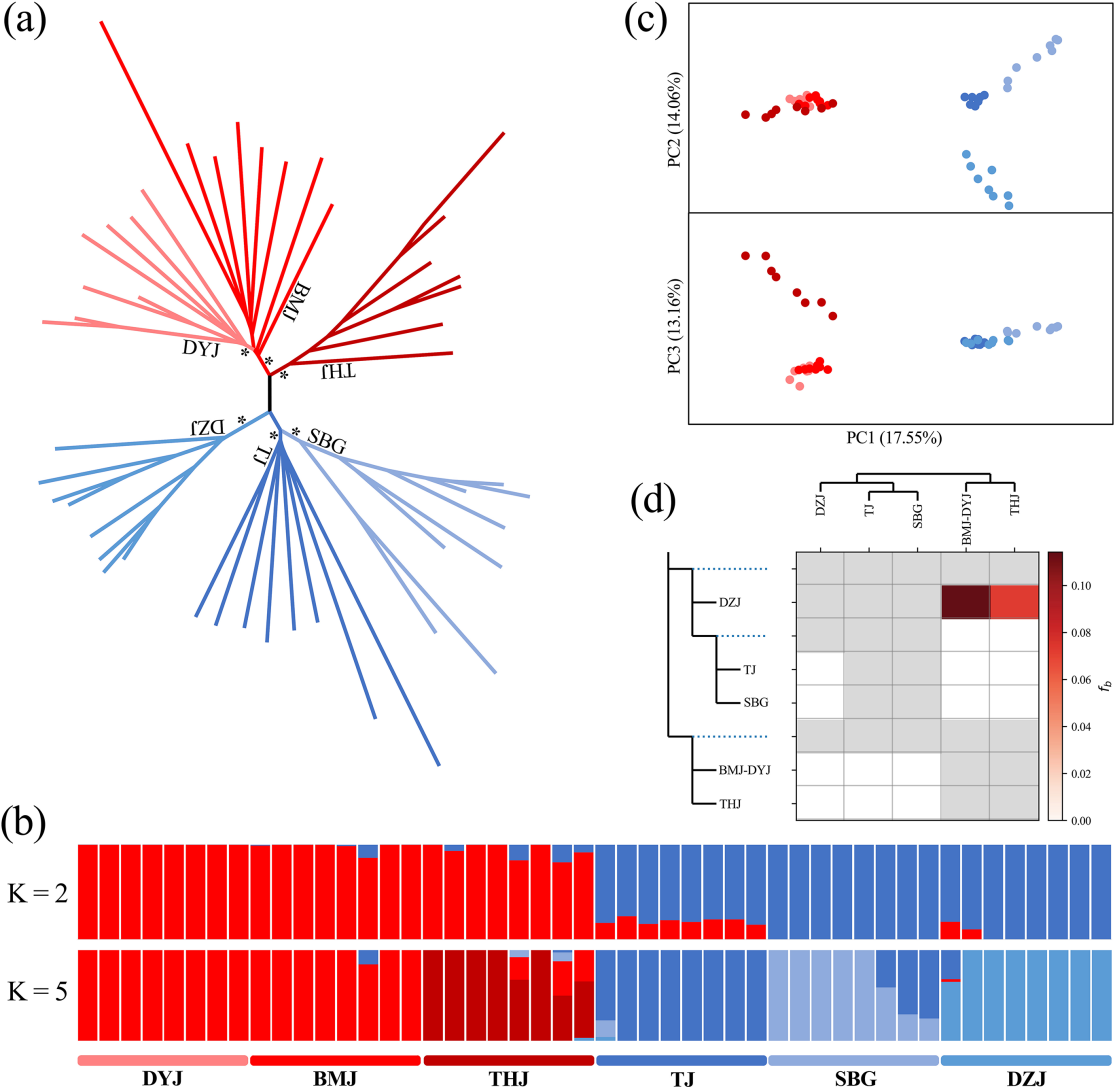
Individual-based genetic structure analyses population-based treemix analysis of *R. shanii*. **(a)** Results of neighbor-joining (NJ) tree clustering, **(b)** ADMIXTURE analysis (K = 2 and K = 5), and **(c)** principal component analysis (PCA), each based on 48 individuals. **(d)** Dsuite analysis for five genetic clusters (in the ADMIXTURE analysis, K = 5) in *R. shanii*. * represents a support rate of 100%.

Whole-genome resequencing of 48 individuals from six *R. shanii* populations generated 772.07 Gb of raw data (mean depth ~26×, **Supplementary Table 2**). The average map rate of these samples of *R. shanii*, *R. griersonianum*, and *R. delavayi* were 95.85%, 96.07%, and 94.02% (**Supplementary Tables 1, 2**). After variant calling and quality filtering, we retained an unlinked dataset of 11,014,032 SNPs for *R. shanii*, averaging 229,459 per individual, which was used for population structure and gene flow analysis. Admixture analysis identified *K* = 2 as the optimal number of genetic clusters for *R. shanii* (**Supplementary Table 3**), consistent with the PCA and NJ tree results (**Figure 2**). These clusters corresponded to two geographic lineages: lineage S (DZJ, SBG, TJ) in the south and lineage N (THJ, DYJ, BMJ) in the north (**Figure 2**; **Supplementary Figure 2**). Notably, all three methods (Admixture, PCA and NJ tree) consistently resolved the six populations into five genetic clusters, with DYJ clustering within BMJ (**Figure 2**). Dsuite analysis detected only limited introgression among populations of *R. shanii* (average *f*-branch statistic = 0.0934; **Figure 2d**). The mean pairwise *F*_ST_ among all populations was 0.057 (**Supplementary Table 4**). *F*_ST_ within lineage S (0.053) was significantly higher than within lineage N (0.035), and both were lower than the mean *F*_ST_ between lineages (0.064). A marginally significant correlation between pairwise *F*_ST_ and geographic distance (r = 0.45, *p* = 0.088; **Supplementary Table 4**; **Supplementary Figure 3**) suggested weak isolation-by-distance.

### 3.2 Genome-wide genetic diversity

**Figure 3:**
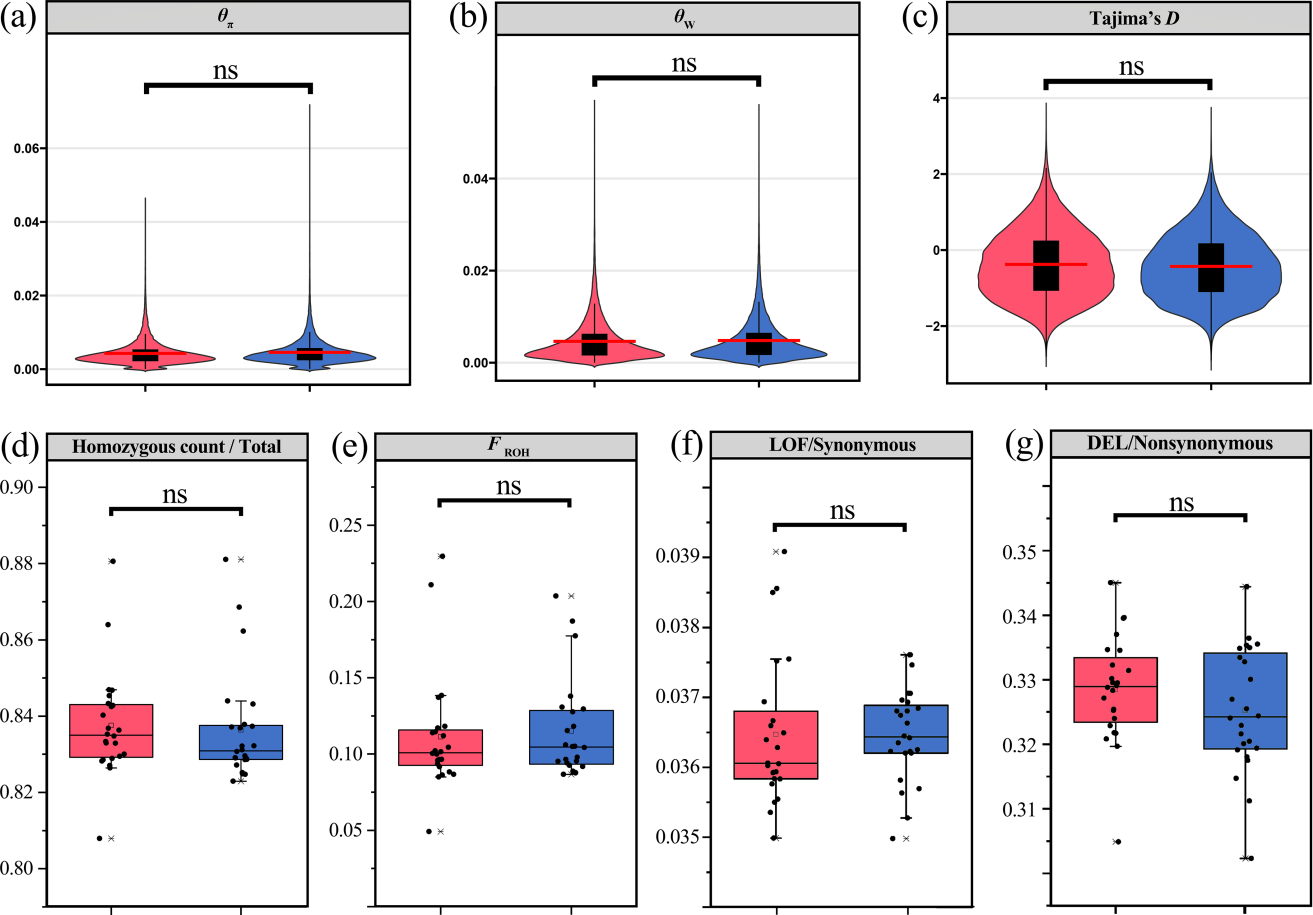
Estimates of inbreeding, genetic diversity, and genetic loads in the two lineages of *R. shanii*. Distributions of *θ*_π_**(a)**, *θ*_W_**(b)**, and Tajima’s *D***(c)** for each lineage calculated in 20-kb windows across the genome. **(d)** genome homozygous ratio and **(e)** estimates of individual genome-based inbreeding (*F*_ROH_) between two lineages. **(f)** Proportion of all genotypes in putatively highly deleterious (LoF) or **(g)** mildly deleterious (DEL) mutations for two lineages. *P*-values of pairwise comparisons were calculated using Wilcoxon test: *P < 0.05; ns, not significant. Red represents lineage N, blue represents lineage S.

**Figure 4:**
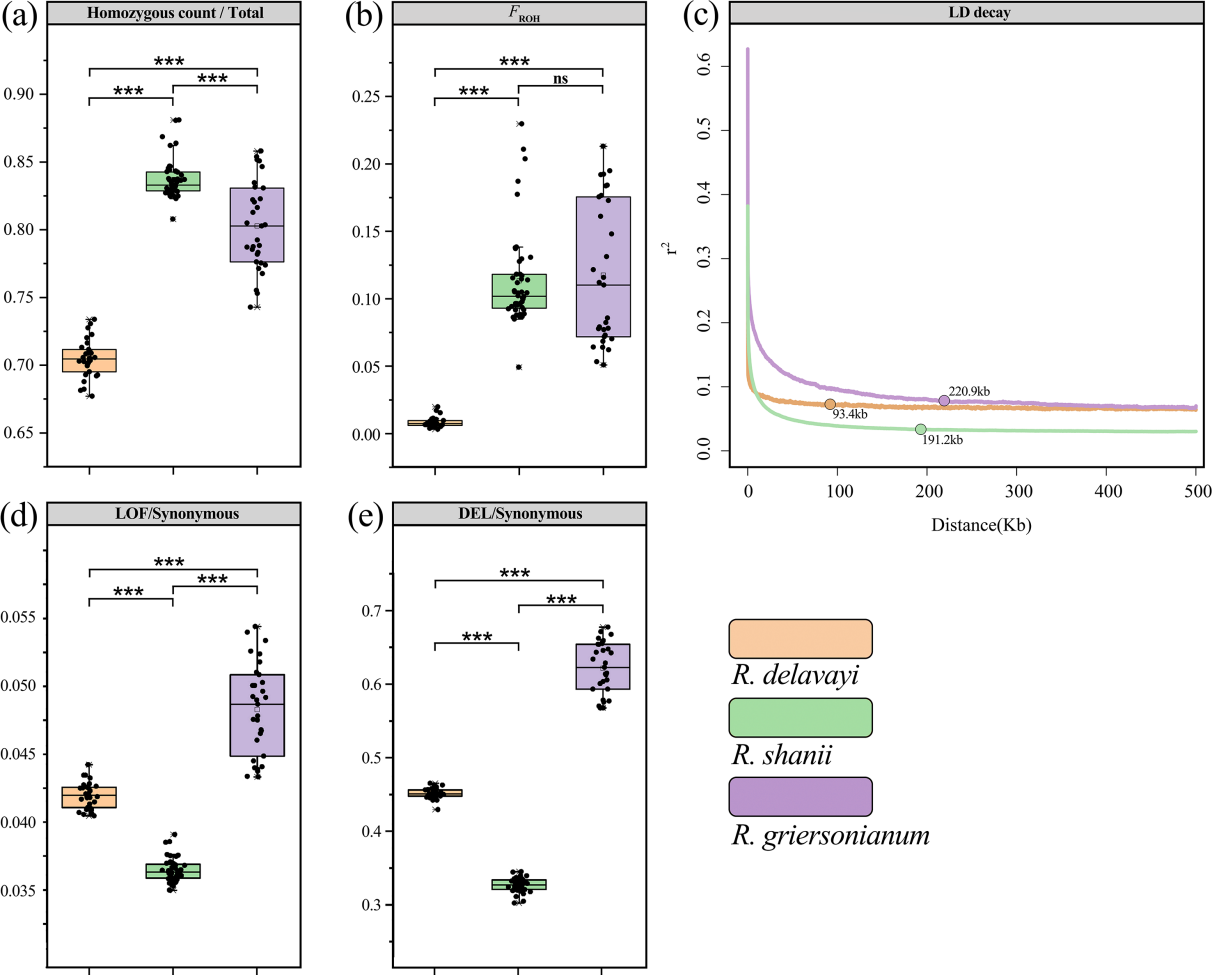
Estimates of inbreeding and genetic loads in *R. shanii* and two related species. **(a)** genome homozygous ratio and **(b)** estimates of individual genome-based inbreeding (*F*_ROH_). **(c)** The decay of LD measured by r^2^. **(d)** Proportion of all genotypes in putatively highly deleterious (LoF) or **(e)** mildly (DEL) mutations. *P*-values of pairwise comparisons were calculated using Wilcoxon test: ***P < 0.001; ns, not significant.

All six populations of *R. shanii* showed similar genetic diversity, with *θ*_W_ ranging from 3.96 – 4.71 × 10–^3^ and *θ*_π_ from 4.20 – 4.64 × 10^-3^ (**Supplementary Table 5**). The mean genomic inbreeding coefficient based on Runs of Homozygosity (*F*_ROH_, 0.10 – 0.13) and Linkage Disequilibrium (LD) decay rates did not differ significantly among populations (**Supplementary Figure 4**; **Supplementary Table 6**). These genetic diversity indices (*θ*_W_ and *θ*_π_), homozygosity metrics (homozygous ratio and *F*_ROH_) and Tajima’*D* values were broadly similar between lineage S and **N** (**Figure 3**). When compared to related species, *R. shanii* (mean *θ*_W_ = 5.45 × 10^-3^, mean *θ*_π_ = 4.87 × 10^-3^) exhibited lower diversity than the widespread *R. delavayi* (the mean *θ*_W_ = 11.61 × 10^-3^, mean *θ*_π_ = 12.97 × 10^-3^) but higher than the endangered *R. griersonianum* (mean *θ*_W_ = 2.58 × 10^-3^, mean *θ*_π_ = 1.94 × 10^-3^). *R. shanii* (Tajima’s *D* = -0.650) and *R. griersonianum* (Tajima’s *D* = -0.895) had significantly negative Tajima’s *D* values, whereas *R. delavayi* showed a positive value (Tajima’s *D* = 0.428; **Supplementary Table 5**). Genome-wide heterozygosity and inbreeding in *R. shanii* (homozygous ratio = 0.837; *F*_ROH_ = 0.113) were comparable to those of *R. griersonianum* (homozygous ratio = 0.803; *F*_ROH_ = 0.118), and significantly higher than in *R. delavayi* (homozygous ratio = 0.705; *F*_ROH_ = 0.008; *P* < 0.001; **Figure 4**; **Supplementary Table 7**). LD decay in *R. shanii* and *R. griersonianum* was slow (r² attained equilibrium at ~191.2 kb and ~220.9 kb, respectively), whereas *R. delavayi* exhibited faster decay (equilibrium r² at ~93.4 kb; **Figure 4**).

### 3.3 Demographic and divergence histories

**Figure 5:**
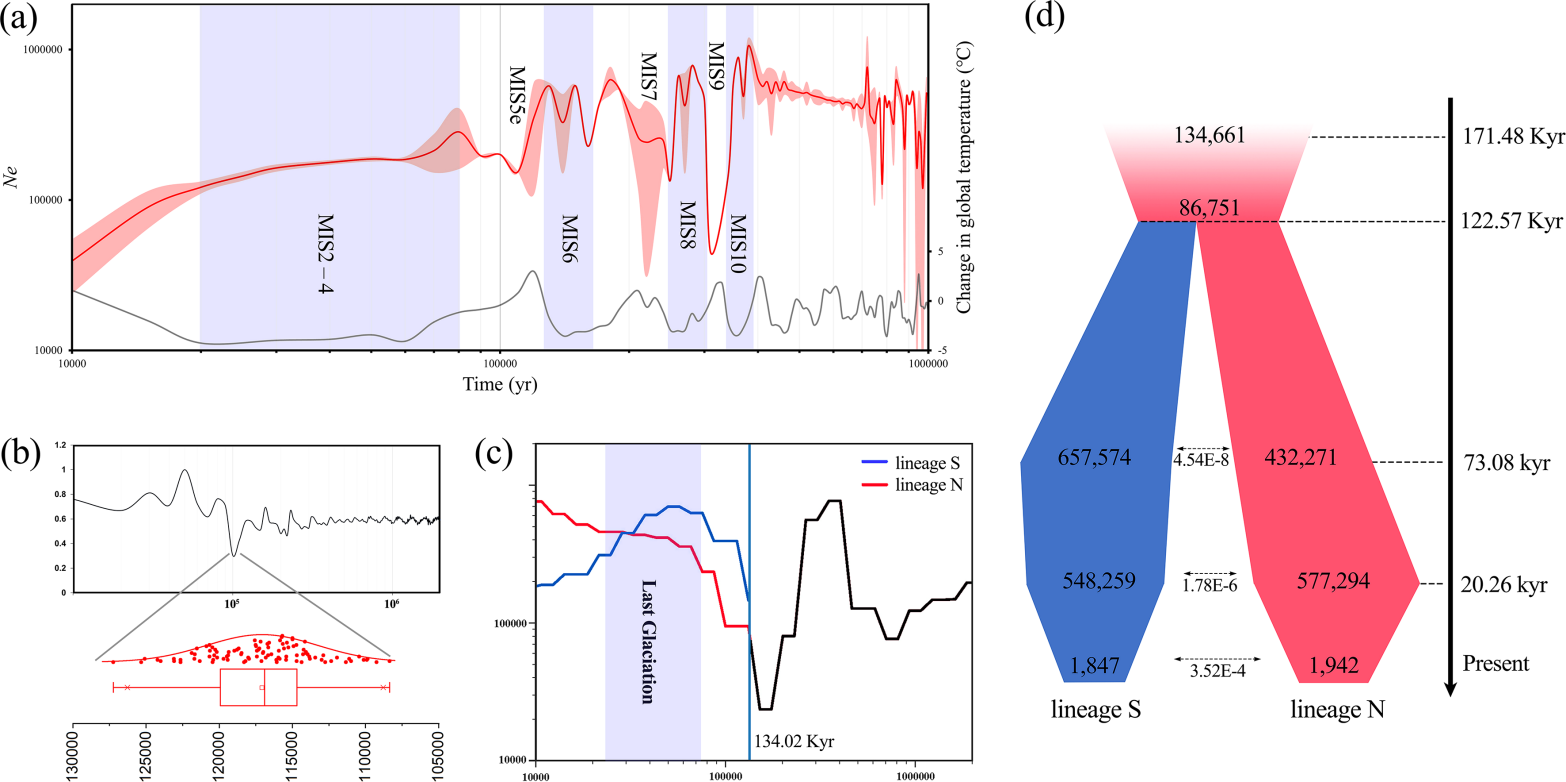
Demographic history of *R. shanii*. **(a)** Effective population size (*Ne*) through time plot using FitCoal for *R. shanii*, pink represents the error value. Marine isotope stage (MIS) represents different periods (Cui et al., 2012). **(b)** Divergence timing between two *R. shanii* lineages was estimated using MSMC2. **(c)** Effective population size and divergence timing between two *R. shanii* lineages was cross-validated using SMC ++ . **(d)** Demographic scenarios modeled for *R. shanii* using fastsimcoal2, with median times in years, as well as estimates of *Ne* and migration rates.

FitCoal demographic analysis identified four sequential population bottlenecks in *R. shanii* over the past 0.4 million years (Mya), each coinciding with interglacial periods (**Figure 5a**). The earliest occurred at ~0.31 Mya (Marine Isotope Stage, MIS 9), followed by events at ~0.21 Mya (MIS 7) and ~0.12 Mya (MIS 5e). The most recent decline began in the Holocene (~0.02 Mya) and has continued to the present, reducing the effective population size (*Ne*) to ~4×10^4^ (**Figure 5a**; **Supplementary Table 8**). Both PSMC (**Supplementary Figure 5**) and Stairway Plot (**Supplementary Figure 6**) analyses consistently revealed population expansions during the Last Glacial Maximum (LGM). Furthermore, Stairway analyses also detected recurrent declines synchronous with both the postglacial Holocene and the Riss-Last Interglaciation (**Supplementary Figure 6**).

Both MSMC2 and SMC++ analyses dated the divergence between the N and S lineages of *R. shanii* to the Last Interglacial (LIG; 0.12–0.13 Mya; **Figures 5b, c**). Furthermore, SMC++ revealed post-divergence expansions in *Ne* for both lineages during cooler periods (**Figure 5c**). We evaluated 15 alternative divergence models using fastsimcoal2, incorporating scenarios with isolation, gene flow, expansions, and bottlenecks (**Supplementary Table 9**). The best-supported model (Model K, AIC = 15,800,316, Δ = 460,758) inferred significant bottlenecks occurred and a lineage split at ~0.12 Mya (95% highest posterior density = 0.10 – 0.14 Mya; **Figure 5d**). Following divergence, both lineages expanded in size as temperatures cooled, but their *Ne* declined steadily during the Holocene. Notably, the lineage S began to decline gradually during the glacial period, whereas the N lineage remained stable until after the LGM. These contrasting demographic trajectories were also supported by SMC++ reconstructions (**Figure 5c**). Low but persistent bidirectional gene flow was detected between the two lineages throughout multiple post-divergence stages (**Figure 5d**).

### 3.4 Genetic load

To explore the patterns of genetic load, we quantified the proportion of the putative loss-of-function (LoF, strongly deleterious mutations) and mildly deleterious (DEL) variants relative to synonymous sites in *R. shanii* and two related species (*R. delavayi* and *R. griersonianum*) using SnpEff and SIFT4G. Across all 48 *R. shanii* individuals, we identified 255,500 LoF sites and 2,289,939 DEL sites (**Supplementary Tables 10, 11**). Within *R. shanii*, the S and N lineages did not differ significantly in the DEL/synonymous and LoF/synonymous ratio (*P* < 0.05, Kruskal-Wallis tests, **Figure 3**). The LoF/synonymous and the DEL/synonymous ratios in *R. shanii* were lower than those of the widespread *R. delavayi* and the endangered *R. griersonianum* (*P* < 0.001, Kruskal-Wallis tests; **Figure 4**).

### 3.5 Species distribution models

**Figure 6:**
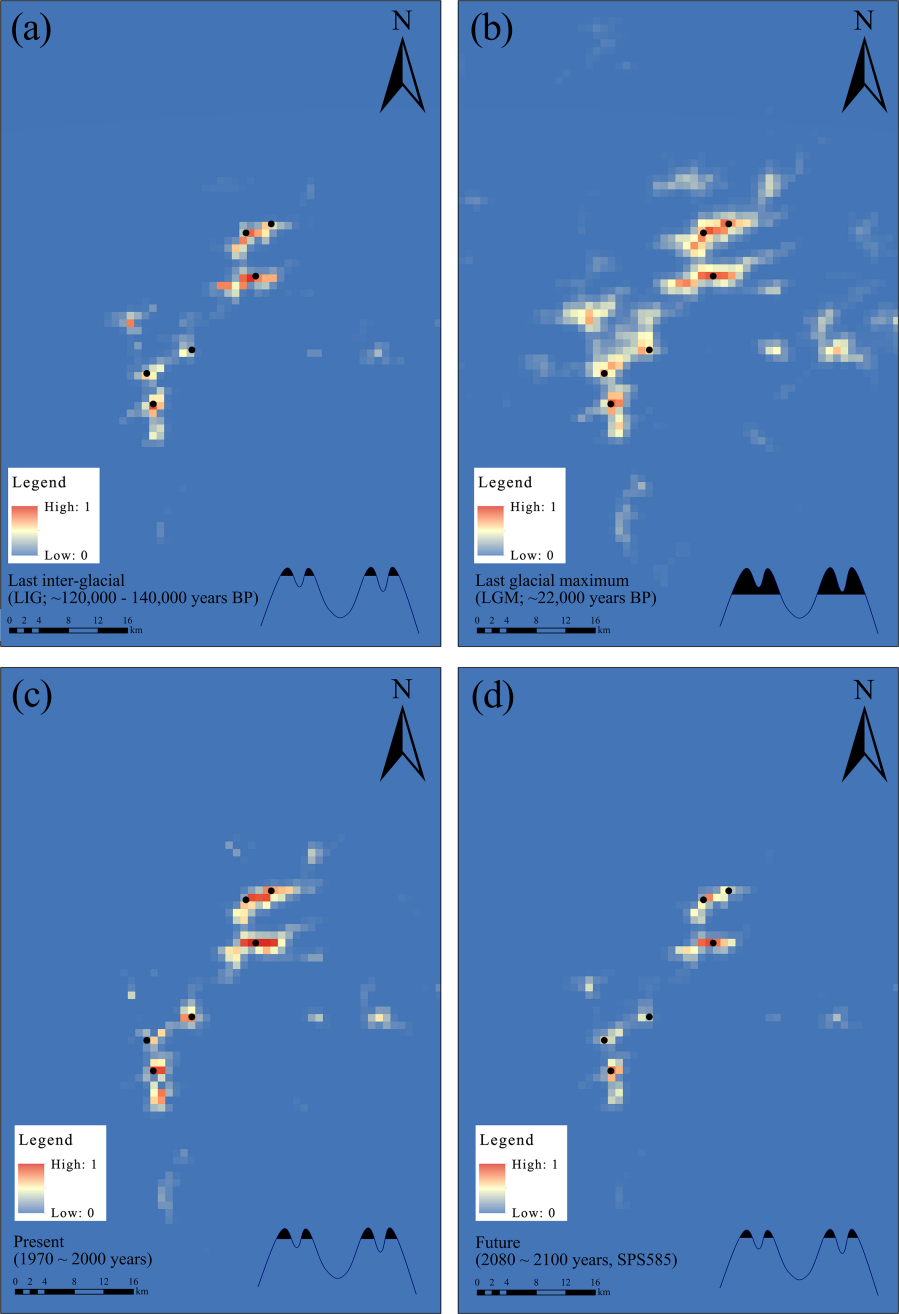
Potential distribution area of *R. shanii* at four periods based on ecological niche modelling using Maxent. **(a)** the last inter-glacial, **(b)** the last glacial maximum, **(c)** the present, and **(d)** the future. The dots indicate the locations of the six populations. The schematic diagram in the lower right corner illustrates the distribution of altitude changes.

We used the MaxEnt algorithm to model the potential distribution of *R. shanii* across four time periods—the Last Interglacial (LIG), Last Glacial Maximum (LGM), current period, and a future scenario (2080–2100, SSP5-8.5). Nineteen bioclimatic variables were evaluated, and jackknife and correlation analyses (**Supplementary Figures 7, 8**) identified three key bioclimatic variables (annual mean temperature, BIO1; mean temperature of driest quarter, BIO9; and precipitation of warmest quarter, BIO18). All simulations exhibited high accuracy (mean AUC = 0.99; **Supplementary Table 12**. During the LIG, the potentially suitable area (suitability > 0.2) was minimal (ca. 56 km²), confined to elevations above ~1,500 m in the Dabie Mountains (**Figure 6a**). By the LGM, suitable habitats expanded to areas ca.171 km^2^ at elevations > ~1,200 m (**Figure 6b**). In the current period, it contracted to only ca. 75 km^2^ above ~1,400 m – closely matching observed occurrence records (**Figure 6c**). Under the future SSP5-8.5 scenario (2080–2100), suitable habitat is projected to further decline to ca. 44 km^2^, restricted to elevations above ~1,600 m(**Figure 6d**). Notably, although the ranges of both the S and N lineages expanded during the LGM, they remained geographically disconnected (**Figure 6b**), suggesting that the habitat fragmentation and isolation have persisted since their divergence.

## 4 Discussion

### 4.1 Glacial expansion and interglacial contraction in *R. shanii*

Although East Asia experienced less extensive Quaternary glaciations than Europe and North America (Harrison et al., 2001; Weaver et al., 1998), Quaternary climatic oscillations also profoundly influenced regional plant population dynamics (Hewitt, 2004; Qiu et al., 2011). Most East Asian woody plants (e.g., *Liquidambar formosana*, *Ostrya chinensis*, *Cercidiphyllum japonicum*, and *Pteroceltis tatarinowii*) underwent significant range contractions during glacial periods, particularly at the LGM. These contractions are followed by notable expansions during interglacial or postglacial periods as temperatures rose (Li et al., 2012; Xu et al., 2024; Yang et al., 2018; Zhu et al., 2020). Strikingly, our results demonstrate a contrasting demographic history for *R. shanii*, characterized by glacial expansion and interglacial contraction. Based on genomic data, FitCoal analysis indicated that this species underwent three distinct population bottlenecks over the past 0.40 Mya, occurring at ~0.12, ~0.20, and ~0.30 Mya, respectively. These events temporally align with the last three major interglacials, i.e., the MIS 5e, MIS 7, and MIS 9 (Cui et al., 2012). Crucially, post-bottleneck population size expansions correlated strongly with subsequent cooling. During the Holocene, the population has declined continuously with rising temperatures (**Figure 5**). Three other analyses (PSMC, Stairway Plot, and Fastsimcoal2) also revealed parallel late-Quaternary population dynamics in *R. shanii*, marked by larger effective population sizes during the LGM and continuous rapid decline during the Holocene. Species ecological niche modeling further demonstrated that *R. shanii* exhibited relatively extensive suitable habitat areas during late-Quaternary cold phases (e.g., the LGM), while habitats contracted substantially during warm periods, including the LIG, Holocene, and future projections.

The glacial expansion and interglacial contraction dynamics observed in *R. shanii* can be mainly explained by its cold-adapted biological traits and limited altitudinal migration capacity within mid-elevation mountain systems.Field surveys confirm *R. shanii* is currently restricted to north-facing slopes above 1500 m in the Dabie Mountains, where it co-occurs with characteristic cold-adapted species, including *Sorbus alnifolia*, *Oyama sieboldii*, *Carpinus viminea*, *Lindera obtusiloba*, and *Quercus stewardii*. This species assemblage and specific habitat requirements indicate highly specialized ecological preferences. Moreover, previous repeated attempts to transplant some seedlings to the conservation station's botanical garden (~1200 m a.s.l.) have consistently failed (personal communications), demonstrating the ecological amplitude of *R. shanii* was exceptionally narrow. Such cold-adapted plants frequently exhibit glacial expansion/interglacial contraction population dynamics (Espíndola et al., 2012; Huang et al., 2023). While many cold-adapted plants can track suitable habitats through altitudinal migration during climatic shifts, the limited elevation shift (mid-elevation topography) of the Dabie Mountains severely restricts this adaptation pathway. With its current distribution already confined to summit and near-summit areas, further warming will accelerate population contraction. This vulnerability is evidenced by notably smaller census sizes observed at the two lower-altitude sites, SBG and DZJ (**Figure 1**; **Table 1**). Thus, limited altitudinal migration space, combined with Quaternary climate oscillations, drives the pronounced historical population fluctuations of *R. shanii*.

As the largest genus of woody plants in the Northern Hemisphere, *Rhododendron* constitutes a key component of montane ecosystems and serves as a classic model for studying alpine adaptation and species differentiation. Currently, the Himalaya Hengduan Mountains and Southeast Asia are recognized as its primary centers of species diversity and endemism (Chamberlain et al., 1996). Most species within this genus are typically adapted to cold environments. Studies have shown that high-temperature stress reduces chlorophyll fluorescence and content, while RdbHLH153 and RdMYB1R1 have been identified as enhancing heat tolerance by decreasing the concentrations of H_2_O_2_ and O^-_2_^ in *Rhododendron* (Khan et al., 2025; Wang et al., 2025). Demographic history reconstructions suggest that *R. shanii* underwent glacial expansion and interglacial contraction, further supporting its classification as a heat-sensitive species, similar to most other members of the genus. Elucidating these heat-sensitive molecular mechanisms will provide valuable insights for the artificial propagation and selective breeding of *Rhododendron* species.

### 4.2 Divergence and demographic history related to climate change

The current distribution of *R. shanii* is extremely restricted, confined solely to the heartland of the Dabie Mountains within an area less than 400 km^2^ (Zhao et al., 2012). Its habitat is limited to six discrete mountaintops above 1500 m a.s.l., totaling less than 300 hectares (**Figure 1**; **Table 1**). At these high elevations, populations are isolated from one another by unsuitable lower-elevation terrain due to niche conservatism. This creates geographic barriers to gene flow, resulting in habitat fragmentation analogous to oceanic islands isolated by seas—hence referred to as "sky islands" (Heald, 1951; He and Jiang, 2014). Species inhabiting such montane sky islands often exhibit high levels of inter-population genetic divergence and unique genetic structures (Chen et al., 2019; Wu et al., 2022), patterns particularly strong in mid- to high-altitude mountains of mid-latitude regions (Pan et al., 2019b; Zhu et al., 2011). Genomic data indeed reveal significant genetic divergence among *R. shanii* populations, with six populations clustering into five distinct groups despite their narrow distributions (**Figure 2**). An exception is the DYJ population, which shows admixture with the BMJ populations due to their proximity and connection via a saddle at approximately 1500 a.s.l. (**Figure 1**). Notably, the three southern populations (TJ, SBG, DZJ) and the three northern populations (THJ, BMJ, DYJ) have diverged into two distinct lineages (S and N). Analyses using three independent methods (MSMC2, SMC++, Fastsimcoal2) consistently date the divergence of lineages S and N during the LIG period (about 0.12–0.13 Mya). Species ecological niche modeling indicated that elevated interglacial temperatures compressed populations into refugia near the summits of northern and southern mountain ranges (**Figure 6**). This range contraction reduced effective population size, intensifying genetic drift and accelerating divergence (Ellstrand and Elam, 1993). Following this initial divergence, both effective population sizes and distribution areas recovered substantially (**Figures 5**, **6**). Nevertheless, secondary contact between the two lineages remained unlikely, as neither Fastsimcoal2 nor Dsuite analyses detected significant gene flow between them (**Figures 2d**, **5d**). Furthermore, ecological niche modeling also showed that suitable habitats for lineages S and N remained disjunct even during the LGM—the period of maximum habitat extent (**Figure 6b**). During the Holocene, climate warming drove rapid declines in the effective population sizes of both lineages, though lineage N declined later than lineage S (**Figure 5**). This temporal disparity likely reflects the marginally higher latitudes of northern populations, demonstrating climate change's profound impact on *R. shanii*. Species in high-altitude mountains or high-latitude regions may possess sufficient geographic space to expand during glacial periods and contract during warm periods (Hu et al., 2013; Huang et al., 2023). In contrast, the mid-elevation *R. shanii* has already compressed its suitable habitat to mountaintops during warm periods, forming isolated summit refugia and thereby reducing its resilience to climate change. This pattern aligns with the phalanx model observed in other species in the region under climatic stress, where complex topography and climatic fluctuations jointly shape this predictable pattern of genetic differentiation (Qiu et al., 2011). In summary, historical climatic fluctuations interacting with island-like summit habitats have driven lineage dynamics and differentiation in this species.

### 4.3 Genetic diversity and genetic loads

Compared to widespread plant species, the genetic diversity of narrowly distributed species is likely to be lower due to inbreeding depression and genetic drift (Ellegren and Galtier, 2016; Feng et al., 2024; Jing et al., 2023). Although *R. shanii* has a remarkably limited distribution range, it harbors relatively high genetic diversity when compared to the other 23 woody plant species for which genetic diversity data derived from whole-genome resequencing were available (**Supplementary Table 13**). Its nucleotide diversity (*θ*_π_) was significantly higher than that of most narrowly distributed and endangered species (e.g., *Ostrya rehderiana*, *C. japonicum* and *R. griersonianum*) and was comparable to, or even slightly higher than, that of relatively widely distributed Asian plants (such as *Liquidambar formosana* and *L. acalycina*) (**Supplementary Table 13**). Generally, wild populations undergoing long-term declines often exhibit low genetic diversity, high levels of inbreeding, and increased genetic load (Feng et al., 2019; Hedrick and Garcia-Dorado, 2016; Wang et al., 2016). As expected, among the three closely related *Rhododendron* species, the endangered *R. griersonianum* exhibited relatively low genetic diversity alongside elevated inbreeding levels and genetic load. In contrast, the widespread *R. delavayi* displayed high genetic diversity with low inbreeding and genetic load (**Figure 4**; **Supplementary Table 10**, **Supplementary Table 11**). Notably, while *R. shanii* showed high inbreeding levels, comparable to *R. griersonianum* as indicated by homozygosity ratio (*F*_ROH_ and LD decay), it maintained relatively high genetic diversity and low genetic load (**Figure 4**). This discrepancy likely stems from their distinct demographic histories. *R. griersonianum* experienced a severe population bottleneck during the LGM (~0.02 Mya, Ma et al., 2021), whereas the last major bottleneck for *R. shanii* occurred during the LIG period (~0.12 Mya). Subsequently, the *R. shanii* population recovered substantially, increasing ~10-fold as temperatures decreased. It maintained this large effective size throughout the LGM but began a rapid decline during the Holocene, continuing to present (**Figure 5**). Species experiencing recent rapid declines in effective population size may retain relatively high genetic diversity and low genetic load, as observed in *Emberiza aureola* (Wang et al., 2016). Thus, although *R. shanii*’s rapid decline has already caused negative effects (e.g., increased inbreeding level), it maintains high genetic fitness—characterized by elevated diversity and low genetic load. This suggests the species is in the early stages of accumulating deleterious genetic effects from its reduced population size.

### 4.4 Threats and conservation recommendations

Understanding how past climatic fluctuations affected the distribution and population dynamics of narrowly distributed species is essential for predicting their responses to ongoing global climate change, which is critical for conservation and management (Hewitt, 2004; Shao et al., 2012). Genomic analyses reveal that *R. shanii* underwent glacial expansion and interglacial contraction, with its population declining continuously since the Holocene as temperatures rose. Ecological niche modeling further indicates that the species occupied a larger suitable range during the LGM, whereas its current suitable habitat is significantly reduced and projected to diminish further. Meta-analyses across taxa show that species distributions are shifting toward higher latitudes (median: 16.9 km/decade) and/or higher elevations (median: 11.0 m/decade) under climate warming scenarios (Chen et al., 2011). However, as a cold-adapted species confined to mountain summits, *R. shanii* lacks sufficient elevational space for upward range shifts. Consequently, limited upward migration potential under climate warming change is the primary driver of its ongoing decline and threatened status.

Based on these research findings, we propose the following conservation strategies: (1) Prioritize protection of its suiSupplementary Table haded habitats near mountain summits. Additionally, experimentally remove some selected individuals of competing trees (e.g., *Pinus taiwanensis*) to create small canopy gaps, as field surveys confirm that *R. shanii* seedlings establish predominantly in light gaps, while mature forests under dense shade lack regenerating seedlings. (2) *In-situ* conservation alone cannot fully mitigate primary threats of *R. shanii*—climate warming and limited elevational migration space. Given that extant populations retain relatively high genetic diversity with low genetic load, we recommend immediate, comprehensive germplasm collection and preservation for potential future population reinforcement. Critically, seeds should be collected as far as possible across all different locations due to significant genetic differentiation already observed among them. (3) To address native habitat constraints, the 'latitude-for-altitude' strategy is proposed for *ex-situ* conservation: transplanting artificially propagated seedlings or wild-collected individuals from high-density regeneration patches (e.g., near-summit areas in THJ) to higher-latitude habitats with analogous companion species (e.g., *Oyama sieboldii*) or botanical gardens.

## 5 Conclusions

By integrating genomic data and species distribution modeling, we identified drivers of genetic structure in *R. shanii*, a mid-elevation sky island species endemic to eastern Asia. Our analyses demonstrate that Quaternary climatic oscillations, coupled with limited altitudinal migration space, drove pronounced glacial expansion and interglacial contraction in the demographic history of *R. shanii*. Warm temperatures during the Last Interglacial triggered its genetic intraspecific divergence. Despite Holocene declines in effective population size leading to increased inbreeding, these populations still maintain relatively high genetic diversity alongside low genetic load. Our findings provide new insights into how past climatic changes affected the demographic history and genetic architecture of cold-adapted plants in the Dabie Mountains, advancing our understanding of adaptive trajectories and conservation strategies for mid-elevation sky island systems organisms under global warming scenarios.

## Data Availability

The datasets presented in this study can be found in online repositories. The names of the repository/repositories and accession number(s) can be found in the article/**Supplementary Material**.
