# Supplementary material for: Quaternary climatic oscillations shaped the demographic history and triggered intraspecific divergence of Rhododendron shanii, a mid-montane endemic in eastern Asia
Source: Front Plant Sci. 2026 Jan 12;16:1740252. doi: 10.3389/fpls.2025.1740252 (PMC12833463; doi:10.3389/fpls.2025.1740252)
Supplement: Supplementary file 2 [file DataSheet1.docx]

**Supplementary data for**

**Title:** Quaternary climatic oscillations shaped the demographic history and triggered intraspecific divergence of *Rhododendron shanii,* a mid-montane endemic in eastern Asia

Yong Deng^1,2^, Zhen Li^1,3^, Yingfeng Hu^1^, Zhizhong Li^4^, Siyu Zhang^5^, Kun Liu^1,3^, Jianwen Shao^1,3*^

^1^College of Life Sciences, Anhui Normal University, Wuhu 241000, Anhui, China

^2^Anhui Forest Survey and Planning Institute, Hefei 230088, Anhui, China

^3^The Anhui Provincial Key Laboratory of Biodiversity Conservation and Ecological Security in the Yangtze River Basin, Anhui Normal University, Wuhu 241002, Anhui, China

^4^Collaborative Innovation Center of Recovery and Reconstruction of Degraded Ecosystem in Wanjiang Basin, Anhui Normal University, Wuhu 241002, Anhui, China

^5^College of Civil and Architecture Engineering, Chuzhou University, Chuzhou 239000, Anhui, China

**^*^ Corresponding author:**

Jianwen Shao

Email: [shaojw@ahnu.edu.cn](mailto:shaojw@ahnu.edu.cn)


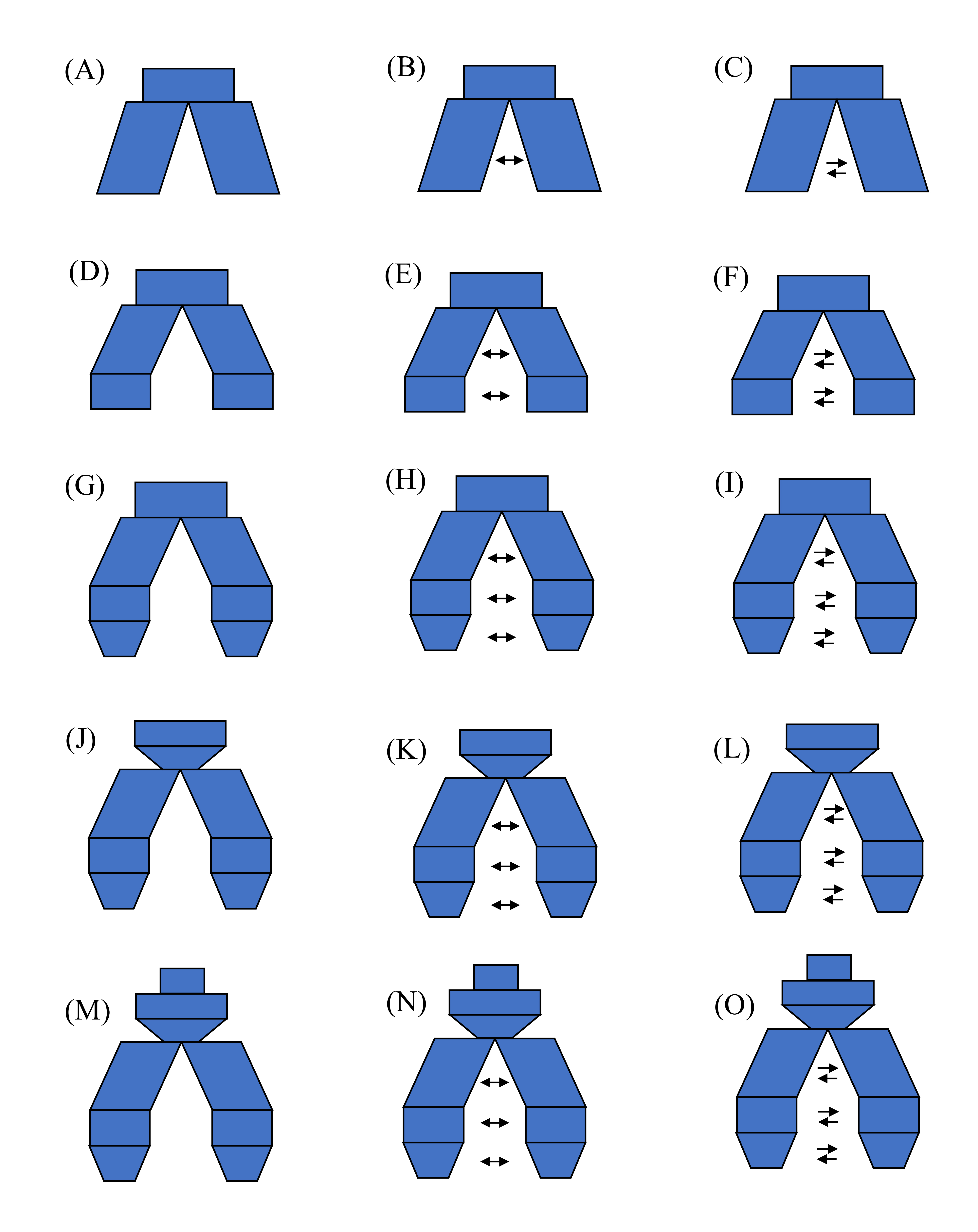


**Supplementary Figure 1.** Scenarios for the divergence and demographic history of the two lineages of *R. shanii*.


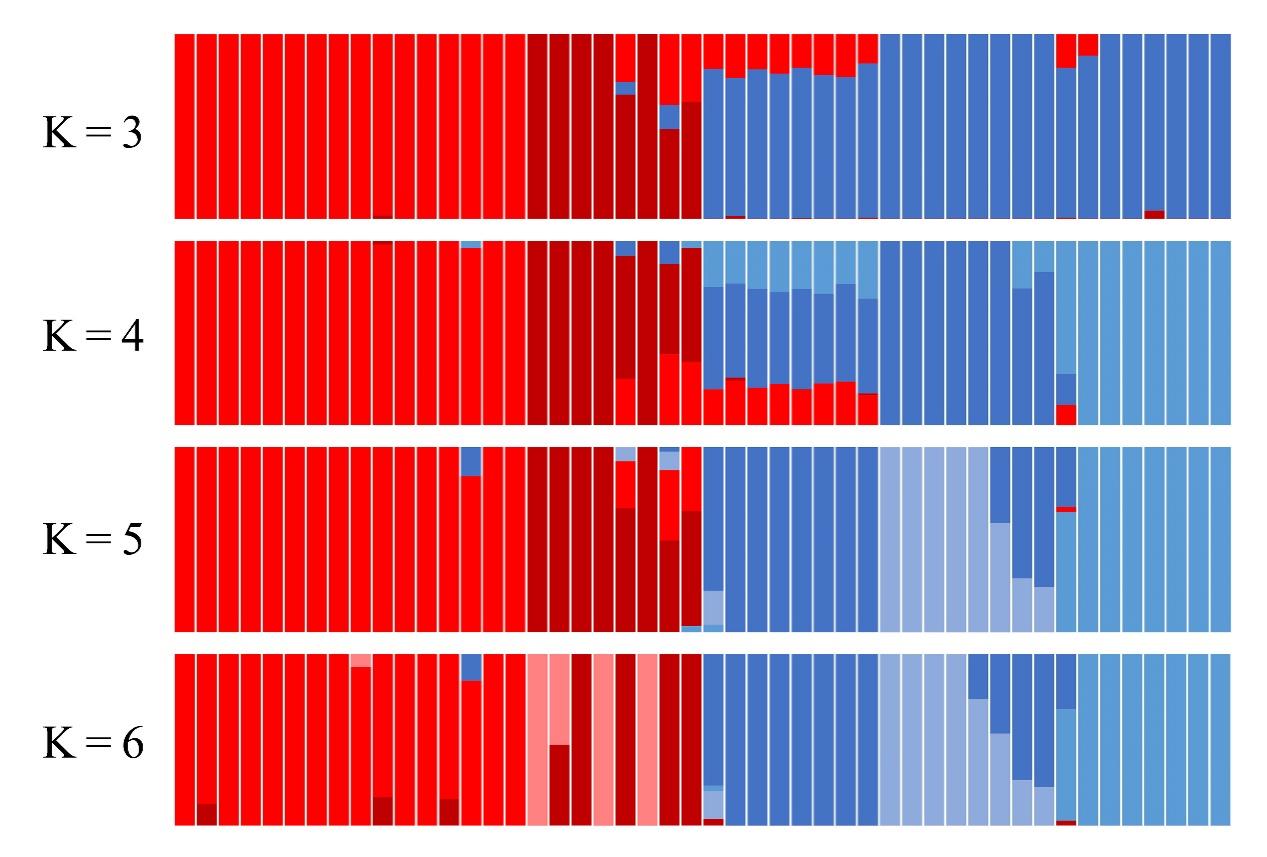


**Supplementary Figure 2.** Result of ADMIXTURE analysis (K = 3 - 6) of *R. shanii*.


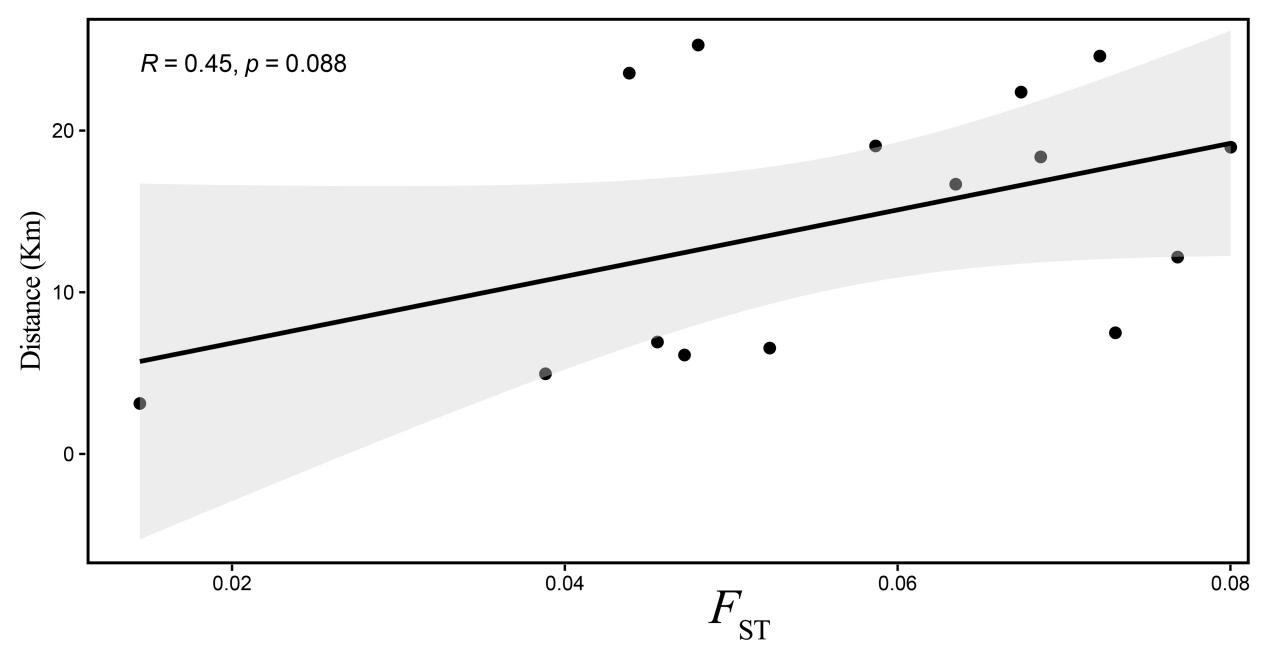


**Supplementary Figure 3.** Result of the correlation between pairwise *F*_ST_ and geographic distance.


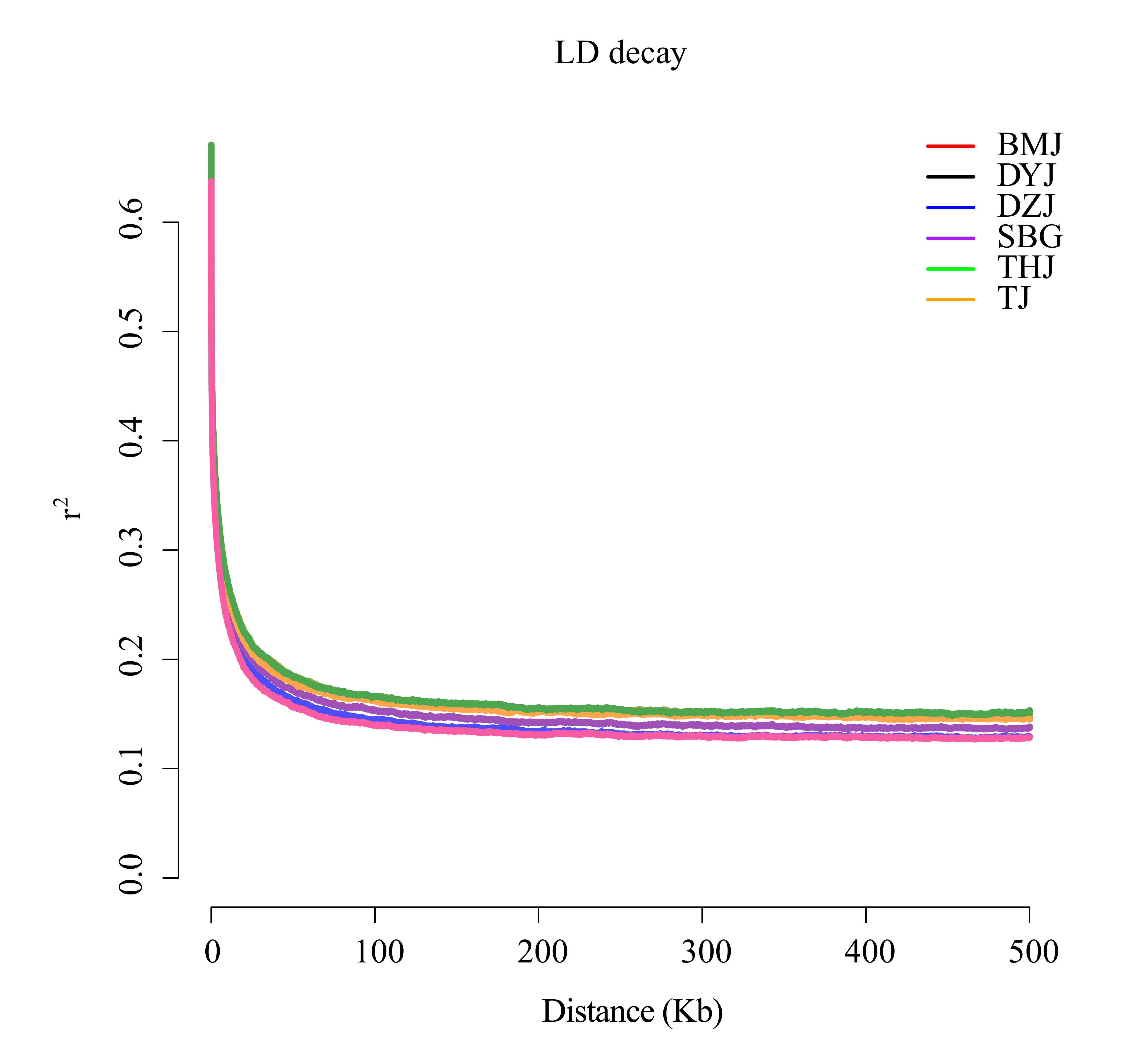


**Supplementary Figure 4.** The decay of LD measured by r^2^ for the six populations of *R.shanii*.


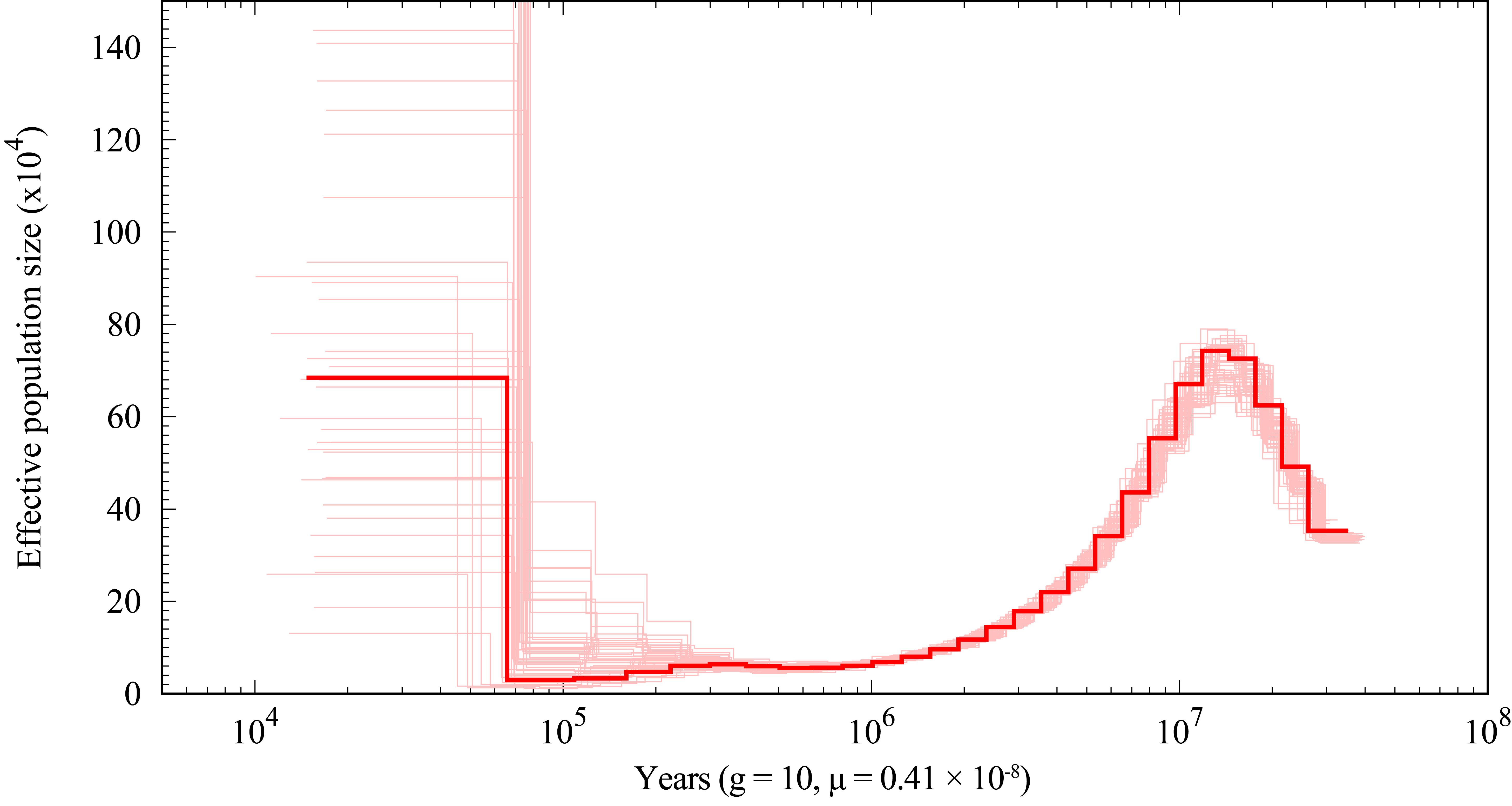


**Supplementary Figure 5.** Changes in historical population size (*Ne*) inferred by the pairwise sequentially Markovian coalescent (PSMC) model for all individuals of *R. shanii*.


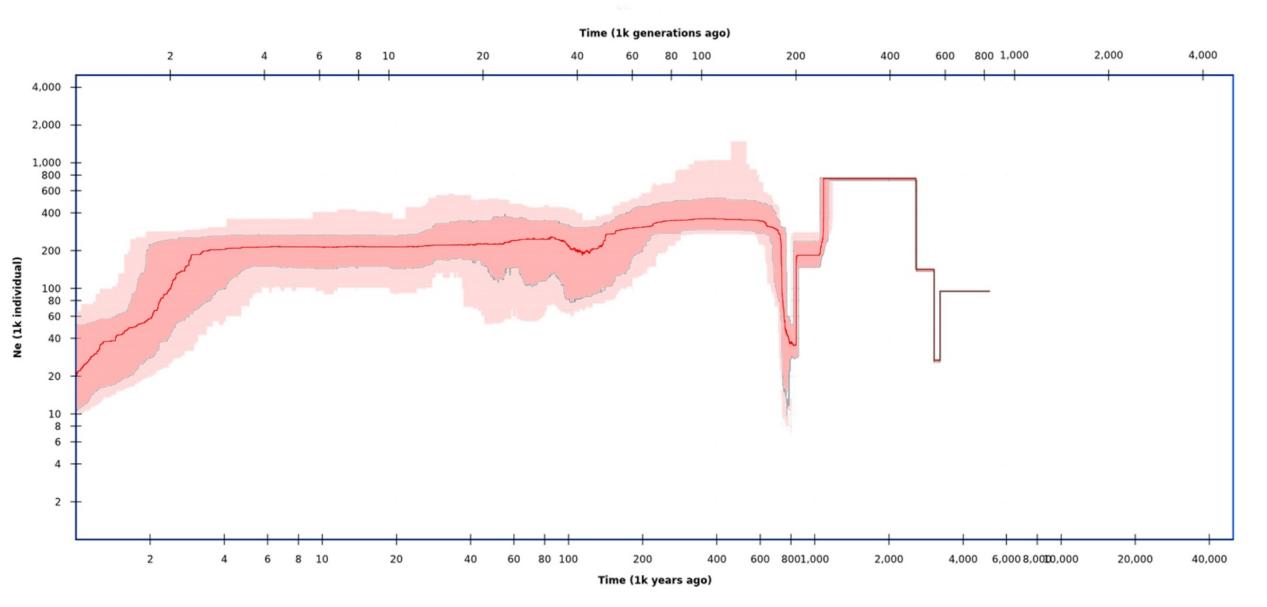


**Supplementary Figure 6.** Results of the STAIRWAY PLOT analyses, illustrating changes in effective population size (*Ne*) through time for *R. shanii*.


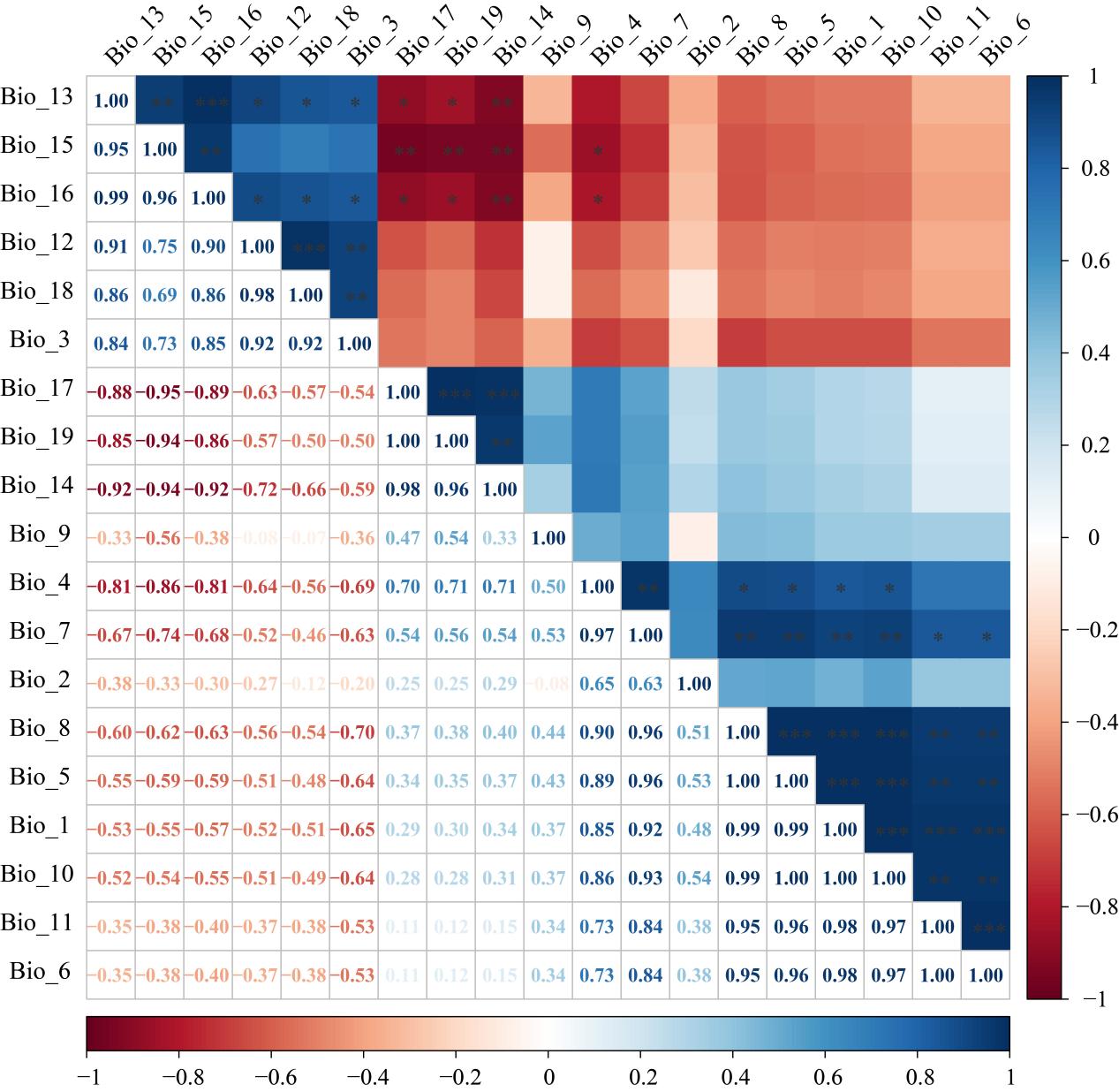


**Supplementary Figure 7.** Correlation analysis of 19 biological factors.


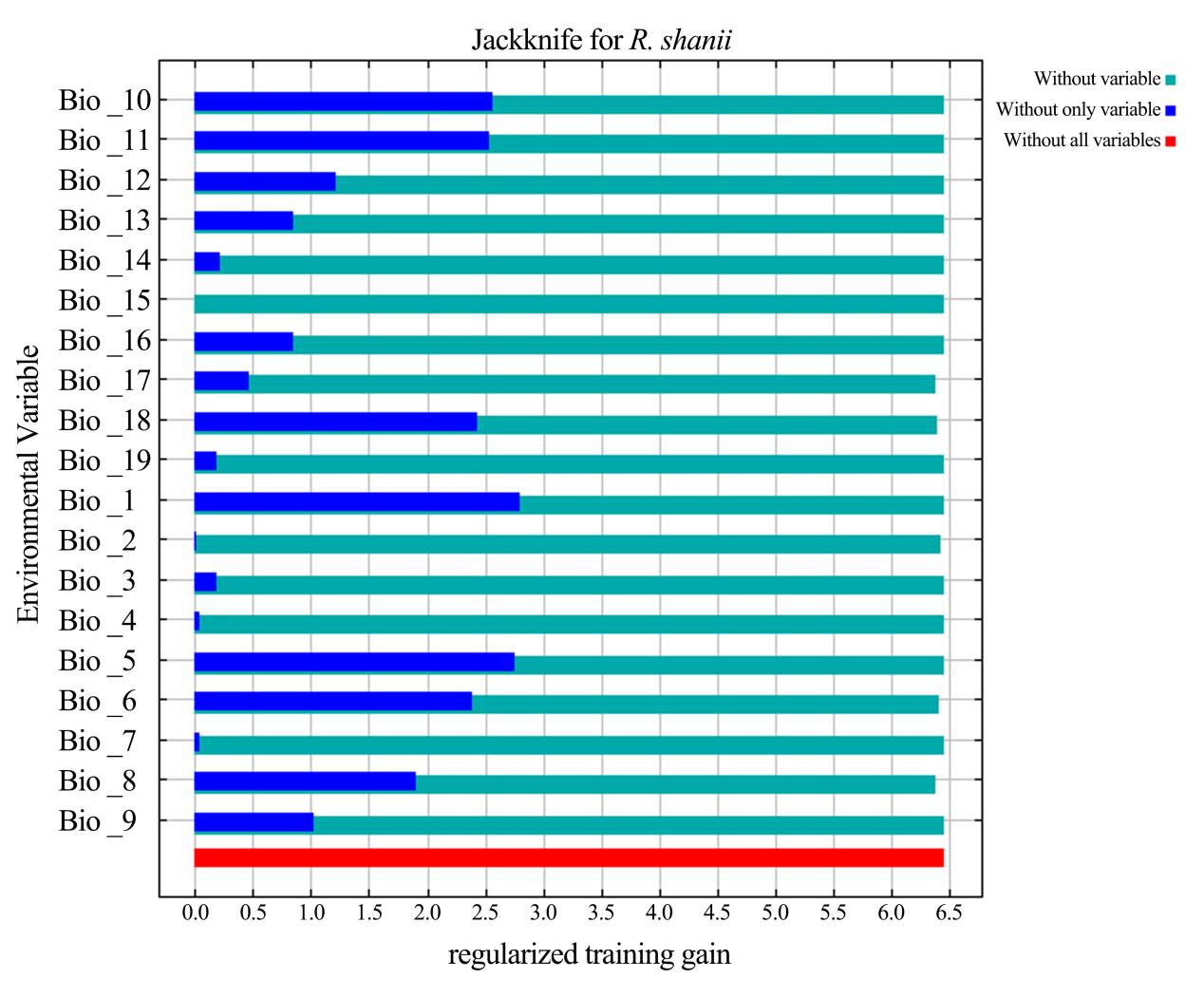


**Supplementary Figure 8.** Jackknife analysis of 19 biological factors.
